# Australian parental perceptions of genomic newborn screening for non-communicable diseases

**DOI:** 10.3389/fgene.2023.1209762

**Published:** 2023-06-26

**Authors:** Sarah Casauria, Sharon Lewis, Fiona Lynch, Richard Saffery

**Affiliations:** ^1^ Murdoch Children’s Research Institute, Melbourne, VIC, Australia; ^2^ Australian Genomics, Melbourne, VIC, Australia; ^3^ Department of Paediatrics, University of Melbourne, Parkville, VIC, Australia; ^4^ Melbourne Law School, University of Melbourne, Parkville, VIC, Australia

**Keywords:** genomic sequencing, newborn screening, non-communicable disease, polygenic risk score, quantitative

## Abstract

**Background:** Newborn bloodspot screening (NBS) programs have improved neonatal healthcare since the 1960s. Genomic sequencing now offers potential to generate polygenic risk score (PRS) that could be incorporated into NBS programs, shifting the focus from treatment to prevention of future noncommunicable disease (NCD). However, Australian parents’ knowledge and attitudes regarding PRS for NBS is currently unknown.

**Methods:** Parents with at least one Australian-born child under 18 years were invited via social media platforms to complete an online questionnaire aimed at examining parents’ knowledge of NCDs, PRS, and precision medicine, their opinions on receiving PRS for their child, and considerations of early-intervention strategies to prevent the onset of disease.

**Results:** Of 126 participants, 90.5% had heard the term “non-communicable disease or chronic condition,” but only 31.8% and 34.4% were aware of the terms “polygenic risk score” and “precision medicine” respectively. A large proportion of participants said they would consider screening their newborn to receive a PRS for allergies (77.9%), asthma (81.0%), cancer (64.8%), cardiovascular disease (65.7%), mental illness (56.7%), obesity (49.5%), and type 2 diabetes (66.7%). Additionally, participants would primarily consider diet and exercise as interventions for specific NCDs.

**Discussion:** The results from this study will inform future policy for genomic NBS, including expected rate of uptake and interventions that parents would consider employing to prevent the onset of disease.

## Introduction

Newborn Bloodspot Screening (NBS) programs have been an essential part of neonatal healthcare globally for more than 50 years (Guthrie and Susi, 1963; Woerner et al., 2021). As of 2015, NBS programs globally can screen for up to 50 conditions, commonly metabolic and/or genetic in nature (Therrell et al., 2015). In Australia, NBS programs employ tandem mass spectrometry as the standard screening approach (Metternick-Jones et al., 2015), with an average uptake rate of over 99% (Australian Government Department of Health and Aged Care, 2023; White et al., 2023). However, newer technologies such as genomic sequencing may be a feasible option for screening in the future as their reliability increases and costs decrease (Preston et al., 2021). Genomic sequencing technologies–encompassing exome and genome sequencing–can sequence a large number of genes implicated in health in parallel (Behjati and Tarpey, 2013), making them the logical next step in such population screening programs.

In cases requiring a diagnosis for critically ill infants, genomic sequencing has resulted in higher diagnostic rates than traditional clinical investigations (Donoghue et al., 2017; Farnaes et al., 2018; Stark et al., 2018). However, sequencing healthy newborns (rather than those already unwell) on a population-wide scale shifts the focus from diagnosis to risk assessment, and from treatment to prevention of onset of conditions. Additionally, sequencing a newborn’s genome can support a precision medicine approach. Precision medicine takes into account various genetic variations and environmental factors of subpopulations to facilitate more effective disease management, and is an alternative to the “one-size-fits-all” approach commonly used in therapeutic medicine (Gameiro et al., 2018).

Many studies globally have assessed the feasibility of introducing genomic sequencing into NBS programs–either as a first-line test or as a follow-up test after initial screening–and have stated its potential clinical utility for a range of metabolic and genetic conditions (Bodian et al., 2016; Park et al., 2016; Smon et al., 2018; Holm et al., 2019; van Campen et al., 2019). However, the feasibility of utilising genomic sequencing in NBS programs to screen for risk of non-communicable diseases (NCDs) has not been investigated. NCDs such as cancer, cardiovascular disease, diabetes, and chronic respiratory conditions, contribute to as much as 74% of deaths worldwide (World Health Organisation, 2022). Most NCDs are characterised as multifactorial or complex diseases, meaning many genes, as well as certain environmental factors such as diet, physical activity, and other socioeconomic factors, can contribute to the onset of disease (Chatterjee et al., 2016). Many studies have shown that early life events can impact an individual’s risk of developing various NCDs. For example, stressors such as malnutrition, physical trauma, air pollution, and an increasing sedentary lifestyle in early childhood can lead to a higher prevalence of NCDs in adulthood (Merrick et al., 2019; Rodriguez-Ayllon et al., 2019; Jaddoe et al., 2020; Nelson et al., 2020). Aside from environmental factors, genetic variants associated with various NCDs continue to be identified through genome-wide association studies (GWAS; Ejtahed et al., 2018; Goodarzi, 2018; Xue et al., 2018), demonstrating that some individuals may have a higher genetic predisposition to developing NCDs compared to others who do not have these associated variants. Implementing preventative measures from early in life may reduce the burden of NCDs in the future (Balbus et al., 2013; Ronto et al., 2018; Juan and Yang, 2020), with additional studies suggesting that early health interventions for children, such as improving diet, increasing physical activity, and reducing stress, can reduce an individual’s risk of developing NCDs (Rodriguez-Ayllon et al., 2019; Jacob et al., 2021; Curioni et al., 2022; Lioret et al., 2023).

Nonetheless, the feasibility of utilizing genomic sequencing on a population-wide level to screen for common NCDs remains a contentious debate. Decisions about which conditions should be included in NBS programs are generally guided by the Wilson and Junger criteria (Wilson et al., 1968) which, among other recommendations, suggest that a condition should only be added to a screening program if it is clinically actionable (Wilson et al., 1968; Johnston et al., 2018). While recent research has suggested the potential clinical and personal utility of genomic NBS (Downie et al., 2021; Armstrong et al., 2022), ethical concerns, such as conflicting interpretations of a child’s best interest (Ross and Clayton, 2019), remain around returning risk information about adult-onset conditions when sequencing children. Some countries, including Australia, have published guidelines for genetic and genomic testing that do not generally recommend testing children and/or disclosing risk information for adult-onset conditions in childhood (Botkin et al., 2015; Vears et al., 2020; Moore and Richer, 2022). However, the introduction of polygenic risk scores to predict an individual’s likelihood of developing certain NCDs in adulthood, could circumvent these guidelines by enabling families to adopt early intervention strategies to reduce their child’s risk of developing NCDs later in life (Khera et al., 2018).

Polygenic risk scores (PRS) are considered to be unchanging across an individual’s lifetime, and may therefore be used to estimate an individual’s lifetime genetic risk of disease. The prevailing view is that early identification of those of highest genetic risk, enables effective targeting of limited resources for disease monitoring, intervention and even prevention. However, for most common conditions, the current discriminative ability is low and the prognostic utility for most conditions remains unclear (Lewis and Vassos, 2020). It is likely that clinical implementation of PRS will be most useful where there is an established approach for intervention, either to treat a condition or even prevent its occurrence. The use of genetic screening in this regard is well established in adult cancers (Antoniou et al., 2008) and is further enhanced through a PRS approach (Mavaddat et al., 2019). However, the utility of PRS in newborn screening remains unclear, as it is likely highly dependent on severity of the disease of interest, age-of-onset, and various environmental and socioeconomic factors. Despite this, PRS-based pre-implantation genetic screening is already available internationally (LifeView, 2023; Virginia Center for Reproductive Medicine, 2023) and newborn PRS screening is being explored in several settings, particularly for type 1 diabetes (Winkler et al., 2019; Sims et al., 2022), giving rise to the possibility of utilising this sort of technology in clinical practice.

Research indicates that Australians have expressed some interest in health-related genomic testing in adulthood (Metcalfe et al., 2018; Metcalfe et al., 2019; Savard et al., 2019). Additionally, studies have shown that Australian parents offered genomic NBS have expressed interest in receiving broader findings relating to the health of their child (Downie et al., 2020). However, despite parents being proxy decision makers for their children, Australian parents’ perceptions of genomic NBS for NCDs have not been explicitly investigated. In this study, we therefore aimed to examine Australian parents’ knowledge and awareness of key concepts such as NCDs, PRS, and precision medicine. Furthermore, we aim to identify the proportion of Australian parents who would consider utilising genomic NBS to predict their child’s susceptibility of developing common NCDs. Finally, we explored the types of intervention strategies that Australian parents would consider in childhood to prevent the onset of these NCDs.

## Materials and methods

### Questionnaire design

The online questionnaire comprised five sections: demographics; knowledge and awareness of NCDs and PRS; personal experience with genetic testing; family history of NCDs; and considerations of genomic NBS for NCDs. Seven NCDs were chosen for the questionnaire and included allergies, asthma, cancer, cardiovascular disease, mental illness, obesity, and type 2 diabetes. These are a subset of the most common NCDs that represent the highest burden of disease internationally, as reported by the World Health Organisation (World Health Organisation, 2022). The questionnaire was modelled on a previous study assessing public perceptions of epigenetic concepts (Lynch et al., 2022), and the questions were adapted to inform opinions of genomic NBS for NCDs. Between 29 and 57 questions were shown to participants, depending on answers provided throughout the questionnaire. The full questionnaire can be found in the Supplementary Material.

A draft version of the questionnaire was piloted with researchers and students with a background in genetics, who provided feedback on questionnaire content, layout, and readability. Pilot responses were not included in the final analysis.

### Recruitment

Individuals over the age of 18 who were parents of at least one Australian-born child under the age of 18, who could be reached via social media, and were able to read and understand written English, were eligible to participate in this study. Participants were recruited via an advertisement on various social media pages including the Facebook and Twitter pages of the Murdoch Children’s Research Institute, and online parenting forums. The study team chose to exclude non-parents from this study, and parents whose children were over the age of 18, as they were unlikely to have had participated in recent NBS programs. Eligibility was determined by a set of screening questions at the beginning of the questionnaire. Participation in the questionnaire was anonymous.

### Data collection and analysis

The questionnaire was developed and administered using REDCap electronic data capture tools (RRID:SCR_003445; Harris et al., 2009). Responses were collected from July 31st to 23 September 2019. Responses that were incomplete beyond the screening questions were excluded from the final analysis.

Data were analysed using STATA version 14 (RRID:SCR_012763; Statacorp, 2015). Demographic and categorical data underwent basic descriptive analyses to obtain frequencies and percentages of responses. Participant postcodes were re-coded in STATA to their Index of Relative Socio-economic Advantage and Disadvantage (IRSAD) percentage and then grouped into quartiles to reflect their overall socioeconomic ratings as determined by average household income`, economic resources`, education`, and employment opportunities according to the Socio-Economic Index for Areas (SEIFA; Australian Bureau of Statistics, 2018). For all personal and family history questions for NCDs or a genetic condition, participants who had reported a personal or familial diagnosis of an NCD or genetic condition were re-coded as having a family history for the respective condition. Participants with no personal or familial diagnosis, or an unknown diagnostic history, were re-coded as having no diagnostic history of the condition. Responses regarding prior awareness and experience of various genetic tests were similarly categorised as “heard of the test and have had it”, “heard of the test but not had it”, and “not heard of this test”. Responses which reported having genetic testing comprised of participants who stated that either themselves or their child had undergone the test. Any “prefer not to say” responses for each question were excluded from analysis. For the purposes of statistical analysis, responses regarding education, marital status, yearly income, and employment status were collapsed into fewer categories.

Two-tailed z-tests of proportion were undertaken to analyse differences in awareness of NCDs, PRS, and precision medicine. Chi-squared tests of independence were performed to investigate any associations between genomic NBS preferences for each NCD and demographic groups. Statistical significance was defined as *p* < 0.05.

## Results

### Participant demographics

The questionnaire received a total of 145 responses. Of those, 19 responses were discarded due to incompleteness, leaving 126 responses for analysis.

The majority of participants identified as female (*n* = 121, 96.0%). The largest proportion of participants were aged between 35 and 44 (*n* = 57, 45.2%), and the mean age of participants was 37.1 years old. The mean number of children under 18 years of age was 1.7, and the average age of participants’ youngest child was 3.6 years, with the largest proportion of youngest children between 1 and 4 years of age (*n* = 59, 46.8%). Sixty participants stated that at least one of their children had a medical problem (*n* = 60, 60.6%), with 21 indicating that this was “moderately serious” (35.0%).

Most participants indicated a postgraduate degree was their highest form of education (*n* = 54, 54.5%) and were employed (*n* = 80, 82.5%). Most were married or living with their partner (*n* = 87, 87.9%) and the largest proportion of participants had a yearly household income of between AUD$75,000-$149,999 (*n* = 42, 46.2%), with the main source of income for coming from wages or a salary (*n* = 93, 94.9%). The majority of participants resided in postal areas within the highest SEIFA quartile (*n* = 71, 57.7%).

Most participants were born in Australia (*n* = 79, 79.8%) and currently resided in the country (*n* = 97, 98.0%). Participant demographics are summarised in Table 1.

**TABLE 1:** Participant demographics.

Demographic	Response	
	n	%
Age group *	*n* = 126	
18–34	52	41.3
35–44	57	45.2
45 and over	17	13.5
Number of children under 18 *	*n* = 126	
1	56	44.4
2	58	46.0
3	12	9.5
Age of youngest child *	*n* = 126	
Under 1 years old	29	23.0
1–4 years	59	46.8
5–9 years	24	19.0
10–14 years	9	7.1
15–17 years	5	4.0
Participant gender	*n* = 126	
Male	5	4.0
Female	121	96.0
Has your child ever had a medical problem?	*n* = 99	
No	39	39.4
Yes	60	60.6
Severity of medical problem	*n* = 60	
Not serious	15	25.0
Mildly serious	14	23.3
Moderately serious	21	35.0
Very serious	10	16.7
Marital status	*n* = 99	
Single	8.0	8.1
Married/Living with partner	87	87.9
Separated/Divorced	4	4.0
Highest level of education (more collapsed)	*n* = 99	
High school/College certificate/Diploma	14	14.1
Undergraduate degree	31	31.3
Postgraduate degree	54	54.5
Main source of household income	*n* = 98	
Wages or salary	93	94.9
Pension or benefit	5	5.1
Yearly household income	*n* = 91	
Less than $75,000	10	11.0
$75,000–$149,999	42	46.2
$150,000+	39	42.9
SEIFA quartile	*n* = 123	
<25% quartile	6	4.9
25%–50% quartile	17	13.8
51%–75% quartile	29	23.6
>75% quartile	71	57.7
Employment status	*n* = 97	
Unemployed	12	12.4
Employed	80	82.5
Student	5	5.2
Country of birth	*n* = 98	
Australia	79	80.6
Other	19	19.4
Country of residence	*n* = 99	
Australia	97.0	98.0
Other	2.0	2.0

### Participants’ awareness of non-communicable diseases, polygenic risk scores and precision medicine

The first section of the questionnaire examined the level of awareness amongst participants of three terms chosen by the study team that reflected the rationale of genomic NBS for NCDs: “non-communicable condition or chronic condition”, “polygenic risk score”, and “precision medicine”. The term “non-communicable disease or chronic condition” was more well-known amongst participants (*n* = 114, 90.5%) compared to “polygenic risk score” (*n* = 40, 31.1%; *z* = 9.56, *p* < 0.001) and “precision medicine” (*n* = 43, 33.9%; *z* = 9.18, *p* < 0.001).

### Participants’ prior experience with genetic testing

Participants were asked about whether they had heard of various genetic tests, and to recount prior experience with these tests either for themselves or for their child (Supplementary Figure S1). Genetic testing during pregnancy was the most well-recognised test as 98.3% (*n* = 118) of participants had heard of the genetic test, and a further 66.7% (*n* = 80) of participants or their children had undertaken the test. Pharmacogenomic testing was the least-known test amongst participants, with only 49.6% (*n* = 59) of participants having heard of it, and only one participant (0.8%) stated that they or their children had had a pharmacogenomic test.

### Participants’ personal or familial history of non-communicable diseases or genetic conditions

**FIGURE 1:**
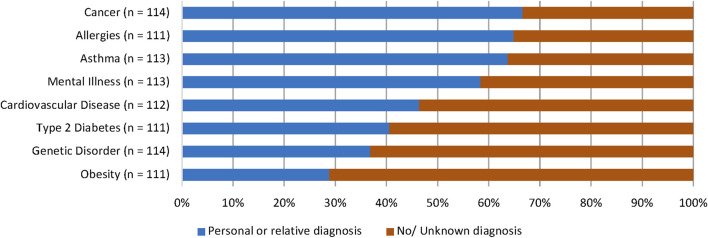
Participants’ personal or familial diagnostic history of NCDs and genetic conditions. Most participants stated that they or a relative had received a diagnosis for cancer (66.7%), allergies (64.9%), asthma (63.7%), and mental illness (58.4%).

Participants were asked to recall any personal or family medical history relating to genetic conditions or common NCDs (allergies, asthma, cancer, cardiovascular disease, mental illness, obesity, type 2 diabetes). The most reported condition that participants or their relatives had received a diagnosis for was cancer (*n* = 76, 66.7%). This was closely followed by allergies (*n* = 72, 64.9%) and asthma (*n* = 72, 63.7%). Obesity had the lowest reported diagnostic history, with 28.8% of participants (*n* = 32) reporting a personal or familial diagnosis. Responses are outlined in Figure 1.

### Consideration of genomic newborn screening for common non-communicable diseases and implementation of health interventions

The third section of the questionnaire asked participants if they would consider genomic NBS for their children to determine their child’s risk of developing seven common NCDs. The largest proportion of participants indicated that they would consider genomic NBS for their child for each respective NCD. Participants’ preferences for genomic NBS across all NCDs are summarised in Table 2.

**TABLE 2:** Participants’ preferences for genomic newborn screening for NCDs.

	NCDs						
	Allergies n (%)	Asthma n (%)	Cancer n (%)	Cardiovascular disease n (%)	Mental illness n (%)	Obesity n (%)	Type 2 diabetes n (%)
Would you consider screening for this condition?							
	*n* = 104	*n* = 105	*n* = 104	*n* = 105	*n* = 104	*n* = 105	*n* = 105
Yes	81 (77.9)	85 (81.0)	68 (65.4)	69 (65.7)	59 (56.7)	52 (49.5)	70 (66.7)
No	20 (19.2)	19 (18.1)	29 (27.9)	28 (26.8)	32 (30.8)	46 (43.8)	32 (30.5)
Don’t know	3 (2.9)	1 (1.0)	7 (6.7)	8 (5.4)	13 (12.5)	7 (6.7)	3 (2.9)
What age would you like to receive results?^a^							
Birth–Age 1	75 (92.6)	73 (85.9)	59 (86.8)	48 (69.6)	42 (71.2)	41 (80.4)	48 (68.6)
2–5	6 (7.4)	10 (11.8)	7 (10.3)	5 (7.2)	5 (8.5)	8 (15.7)	7 (10.0)
6–10	0 (0.0)	1 (1.2)	0 (0.0)	4 (5.8)	10 (16.9)	1 (2.0)	4 (5.7)
11–14	0 (0.0)	1 (1.2)	1 (1.5)	7 (10.1)	2 (3.4)	1 (2.0)	5 (7.1)
15+	0 (0.0)	0 (0.0)	1 (1.5)	5 (7.2)	0 (0.0)	0 (0.0)	6 (8.6)

^a^ Smaller sample sizes as only those who answered “Yes” to screening consideration for the respective NCD, were able to answer the age preference for the provision of results.

Participants who said they would consider genomic NBS for the NCDs presented were then asked about the age at which they would like to receive their child’s result for each specific condition (Table 2). Across all NCDs, most participants preferred to receive results between birth and age 1, irrespective of each condition’s indicative age of onset (as provided to participants in the survey question).

**FIGURE 2:**
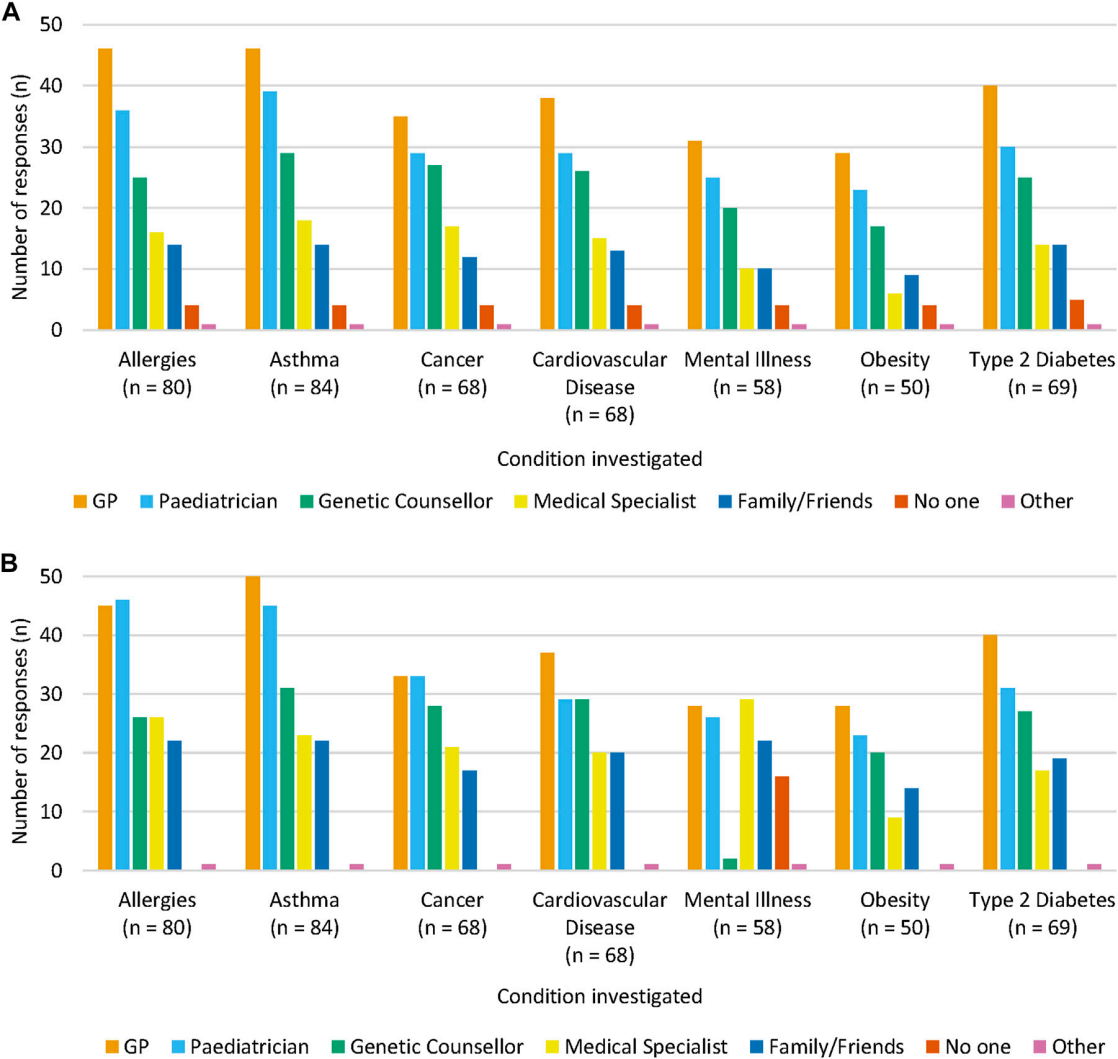
Who participants would prefer to discuss a genomic newborn screening test with **(A)** prior to ordering it and **(B)** who they would discuss results with post-screening test. Participants were able to select more than one answer for each condition, therefore the sum of answer frequencies for each NCD equate to over 100%.

For each NCD, participants were asked who they would want to discuss the screening test with prior to ordering it, as well as who they would want to discuss the results with once received (Figure 2). Participants primarily chose to discuss both pre- and post-test information with a General Practitioner (GP) for asthma (*n* = 46, 54.8% and *n* = 50, 59.5% respectively), cardiovascular disease (*n* = 38, 55.9%; *n* = 37, 54.4%), mental illness (*n* = 31, 53.4%; *n* = 28, 48.3%), obesity (*n* = 29, 58.0%; *n* = 28, 56.0%), and type 2 diabetes (*n* = 40, 58.0%; *n* = 40, 58.0%). For allergies, pre-test discussions were also preferred to be held with a GP (*n* = 46, 57.5%), but most participants preferred post-test discussions to be held with a paediatrician (*n* = 46, 57.5%). For cancer, pre-test discussions were preferred to be held with a GP (*n* = 35, 51.5%), however for post-test discussions, participants equally preferred to speak with a GP or a paediatrician (*n* = 33, 48.5% each). One participant chose “other” for all NCDs when asked who to discuss the post-test results with and explained in a free-text comment that their choice depended on the severity of the NCD.

**FIGURE 3:**
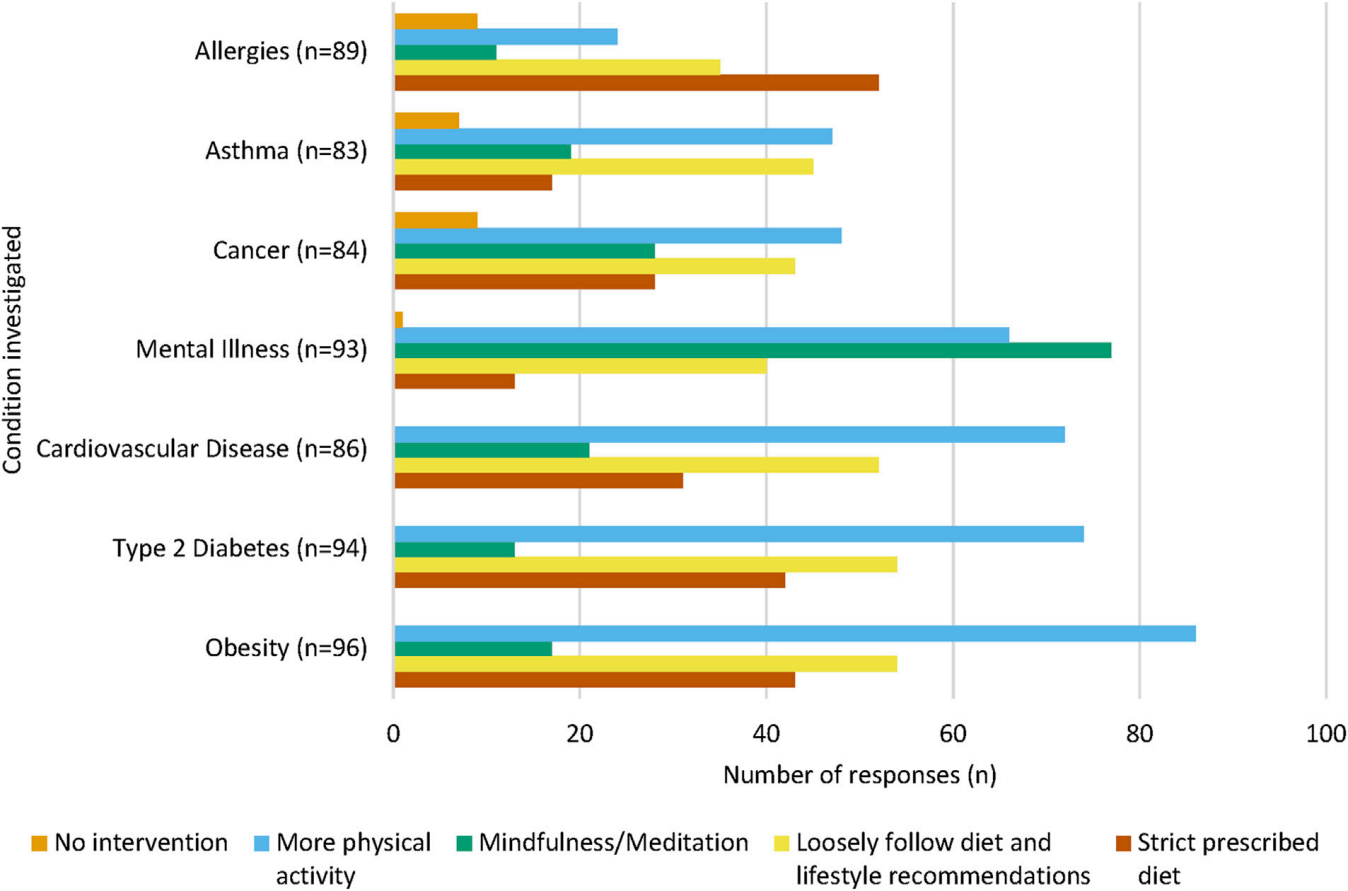
Intervention methods chosen for each NCD. For each NCD, a large proportion of participants chose “More physical activity” as a proposed intervention method. “Mindfulness and meditation” were chosen by the majority of participants to prevent the onset of mental illness. Participants could select multiple interventions for each condition, therefore the sum of each intervention per NCD will equate to over 100%.

At the end of this section of the questionnaire, participants had the opportunity to specify what common interventions they may employ to prevent the onset of each NCD for their children (Figure 3). Increased physical activity was the most common intervention chosen for obesity (*n* = 86, 89.6%), type 2 diabetes (*n* = 74, 78.7%), cardiovascular disease (*n* = 72, 83.7%), cancer (*n* = 48, 57.1%) and asthma (*n* = 47, 56.6%). Most participants chose mindfulness and meditation as an intervention for mental illness (*n* = 77, 82.8%), and a strict prescribed diet was the most commonly intervention strategy for allergies (*n* = 52, 58.4%).

### Effect of participant characteristics on genomic newborn screening preferences for non-communicable diseases

Participants’ personal or familial history of each NCD were not evidenced to influence their preference of genomic NBS for the respective condition (Supplementary Table S1).

Whether or not participants had a child who experienced a medical problem did not show evidence of an effect on their screening preference for any of the NCDs except cancer [(χ2 (2) = 6.388, *p* = 0.041] (Supplementary Table S2), where parents with children who had not experienced a medical problem were more likely to prefer screening for cancer.

Participants’ age showed evidence of an effect on their preferences for screening for allergies [χ2 (4) = 11.485, *p* = 0.022], asthma [χ2 (4) = 15.929, *p* = 0.003], cancer [χ2 (4) = 10.382, *p* = 0.034], mental illness [χ2 (4) = 10.958, *p* = 0.027], and type 2 diabetes [χ2 (4) = 15.942, *p* = 0.003], but did not seem to influence their preferences for screening for cardiovascular disease [χ2 (4) = 9.480, *p* = 0.050] and obesity [χ2 (4) = 1.837, *p* = 0.766] (Supplementary Table S3).

Participants’ education showed evidence of influencing their screening questions for allergies [χ2 (4) = 12.473, *p* = 0.014], asthma [χ2 (2) = 10.736, *p* = 0.005], cancer [χ2 (4) = 14.076, *p* = 0.007], obesity [χ2 (4) = 10.938, *p* = 0.027], and type 2 diabetes [χ2 (4) = 9.568, *p* = 0.048] but did not show evidence of having an effect on their screening decisions for cardiovascular disease [χ2 (4) = 5.387, *p* = 0.250], and mental illness [χ2 (4) = 8.101, *p* = 0.088]. (Supplementary Table S4).

Participants’ SEIFA score based on their residential postcode did not show evidence of influencing their genomic NBS preferences for all NCDs investigated (Supplementary Table S5).

## Discussion

This is the first study to explore Australian parents’ perceptions of genomic NBS, and their awareness of polygenic risk and precision medicine in the context of the NCDs presented. We found that most parents had previously heard about NCDs, but fewer knew about polygenic risk and precision medicine. However, despite the lack of awareness, most parents would consider genomic NBS to provide a PRS at birth for seven common NCDs if it were offered in addition to the current NBS program. Furthermore, the data highlights that parents would prefer to know their child’s risk of developing common NCDs earlier in their child’s life rather than later and would consider undertaking early intervention strategies to reduce their child’s risk of developing NCDs, therefore highlighting the personal and clinical utility of such a screening test.

### Parents know about non-communicable diseases, but not polygenic risk scores or precision medicine

Most participants expressed familiarity with the concept of NCDs, which is comparable to research investigating public awareness and experiences with common NCDs including cancer, diabetes, obesity, and heart disease (Waters et al., 2014). In contrast, participants were largely unaware of PRS and the concept of “precision medicine”, which aligns with the lack of awareness and experience of health-related genomic testing, where PRS and precision medicine concepts may be more commonly discussed. The latter is consistent with a previous Australian study, showing that fewer individuals had undergone genomic tests (such as nutrigenomics and pharmacogenomics tests) compared to more common tests such as genetic carrier screening (Savard et al., 2019).

### Parents value genomic newborn screening

This study showed that a large proportion of participants were in favour of expanding the current NBS program to include seven proposed common NCDs: allergies, asthma, cancer, cardiovascular disease, mental illness, obesity, and type 2 diabetes. Additionally, we found that parents would prefer to know about their child’s risk of developing NCDs earlier in life rather than later, irrespective of the average age of onset for the NCDs investigated.

There is conflicting literature examining parental attitudes around receiving genomic test results for children. Some literature suggests parents want to know their child’s risk of developing a disease in both childhood and adulthood (Tercyak et al., 2011; Kulchak Rahm et al., 2018; Holm et al., 2019; Armstrong et al., 2022) as it is in the child’s–and the family’s–best interest to begin clinical management as early as possible. On the other hand, other studies have demonstrated that parents are hesitant to know about genomic results for later-onset conditions (Sapp et al., 2014; Anderson et al., 2017), with the most common justification being that this type of information could cause additional worry and distress for the family. Nevertheless, our study suggests that parents still find value in genomic NBS for NCDs, highlighting their beliefs of personal utility of this kind of testing, irrespective of the level of information, whether perceived or actual, that a PRS could provide about a child’s health. We found that parents were willing to have their child undergo genomic NBS even if they stated that they had not previously heard of precision medicine or polygenic risk, suggesting that a lack of awareness around the nuances of utilising genomic sequencing to calculate polygenic risk could potentially drive overenthusiasm for its implementation into clinical practice. The notion of the general public’s enthusiasm for genomic sequencing is supported by a similar study, where parents hypothetically opted for genomic newborn sequencing due to the belief that newer sequencing technologies would be more accurate than traditional testing (Dodson et al., 2015). On the other hand, simply expressing interest in genomic NBS to obtain knowledge of their child’s risk status may not reflect the rate of actual participation in an expanded program (Genetti et al., 2018). Further research will be required prior to the implementation of such a program into the Australian healthcare system to investigate any potential discrepancy in anticipated versus actual uptake of genomic NBS.

Furthermore, while research has highlighted the clinical utility of PRS to assess an individual’s genetic predisposition to certain NCDs (Torkamani et al., 2018; Lambert et al., 2019), the observed lack of knowledge around PRS in this study suggests individuals may find PRS information difficult to understand (Naik et al., 2012; Folkersen et al., 2020). How the concept of PRS is communicated to patients will be crucial when considering their implementation. Information regarding the rationale and process of genomic NBS could be given to prospective parents in a similar fashion to the provision of current NBS information. Specifically in Victoria, Australia, parents are not contacted if their child’s screening results are normal, whereas positive screens are followed up immediately with the parents and hospitals (Victorian Government Department of Health and Human Services, 2018). However, further genomics education may be warranted for a parental audience prior to offering any genomic NBS. Additional research may also be required to assess the acceptability of genomic NBS more generally both before and after providing relevant information about the implications of PRS and precision medicine in the neonatal period for later-onset NCDs.

### Influence of perceived risk and severity of non-communicable diseases on parents’ genomic newborn screening preferences

Participants’ opinions of genomic NBS for the seven proposed NCDs in our study did not appear to be significantly influenced by any personal or family medical history of each NCD respectively. This contrasts with previous literature showing that having a family history of a condition can impact parents’ decisions to consider NBS for that condition (Lipstein et al., 2010). The influence of personal or family history of a health condition on an individual’s preference for screening for the condition is explained by the Health Belief Model, which theorises that decision making in healthcare is determined by an individual’s perceived susceptibility and severity of a condition; perceived benefits of a health intervention (such as genomic screening); and perceived barriers to undertaking health interventions (Glanz et al., 2008). These factors combined may provide adequate cues to action that prompt an individual to seek out health interventions (Glanz et al., 2008). Despite this, our findings suggest that an individual’s perceived risk of disease did not significantly influence their decisions to consider genomic NBS for their children. Rather, other factors, not explored in this study, such as perceived severity of disease and perceived benefits of undergoing screening, may have an influence on participants’ considerations for screening.

Perceived severity of disease may also influence an individual’s motivation to adopt early intervention strategies and can therefore inform the development of policies and public health programs to promote healthy lifestyle choices (Azadi et al., 2021). Research has highlighted the importance of NCD prevention and early intervention, noting that consumption of high-calorie foods and a sedentary lifestyle are the two most common risk factors for obesity amongst school-aged children (Motlagh et al., 2017). In the context of polygenic risk, early health intervention strategies have been shown to reduce the risk of developing cardiovascular disease and diabetes in individuals with a higher genetic predisposition to these conditions (Wang et al., 2021). In contrast, other research has shown that genetic risk of NCDs is not necessarily the cause of adopting healthier lifestyle choices for individuals who have undertaken direct-to-consumer genomic testing (Nielsen et al., 2017), rather suggesting the act of undertaking the testing is influencing health behaviour change in individuals (Stewart et al., 2018). In accordance with the latter notion, our study found that Australian parents would largely choose diet and exercise interventions to prevent the onset of all presented NCDs for their child. The idea that the perceived benefits of testing overall are a key influence for parents rather than the actual perceived severity of each disease is important to consider when implementing genomic screening for NCDs into a NBS program. Further exploration is required to determine whether a nationwide genomic NBS program for NCDs (i.e., understanding the genetic predictors of the onset of NCDs), or other health intervention programs that don’t necessarily highlight genetic risk, would be more efficient to promote healthy lifestyle behaviours at an early childhood level.

### Study limitations and future research

Our small study population consisted primarily of well-educated mothers, and many had a child with a medical condition. The questionnaire was shared across social media pages whose audience was mostly well-educated parents, likely with an interest in health research. Therefore, our findings about the attitudes towards genomic NBS are not representative of a diverse population. It is important to be aware of demographic differences amongst health beliefs when providing adequate information for expanded screening programs. For example, knowledge of NCDs and their risk factors, including genetic risk, tends to increase in more highly educated populations (Allen et al., 2017).

While the questionnaire was piloted primarily for the purposes of readability and layout, it was not piloted with a lay audience. This may further limit the transferability of the study findings to the general population. It is crucial that the attitudes of diverse populations regarding genomic newborn screening be appropriately explored before such screening is implemented (Dodson et al., 2015; Genetti et al., 2018). Additional research is needed utilising a more targeted approach to recruit diverse individuals and adequately capture the attitudes of parents from various cultural and socioeconomic backgrounds regarding genomic NBS and its potential outcomes.

Finally, this study aimed to provide an overview of the Australian public’s recognition of terms and phrases related to NBS for NCDs. The limited information in the questionnaire related to this terminology allowed us to examine the public’s superficial understanding of such terms and phrases, however it did not allow us to explore in-depth their accurate comprehension. Further research is needed to examine which terms are best understood by the public, and therefore which are best to use in public health communications.

## Conclusion

This is the first study to examine Australian parental perceptions of genomic NBS for NCDs in the context of polygenic risk and precision medicine. While not representative of the general population, our findings establish a foundation for further research into the Australian public’s perceptions of polygenic risk and precision medicine in the context of NCDs, and considerations of genomic NBS should this type of testing be added to the current NBS program. Future research should focus on expanding the investigation to a more diverse population. Understanding different perspectives around the use of genomic technology in NBS programs will provide valuable information to researchers and policy makers about how to implement an expanded NBS program into the Australian public healthcare system.

## Data Availability

The raw data supporting the conclusion of this article will be made available by the authors, without undue reservation.
