# Supplementary material for: Australian parental perceptions of genomic newborn screening for non-communicable diseases
Source: Front Genet. 2023 Jun 26;14:1209762. doi: 10.3389/fgene.2023.1209762 (PMC10330815; doi:10.3389/fgene.2023.1209762)
Supplement: Supplementary file 1 [file Table1.DOCX]

**Supplementary Table 1.** Effect of participants’ personal or familial diagnostic history on screening preferences for NCDs.

| **Testing Preference** | **Diagnostic history of condition** | | | | | | **Total** | | **chi2(df)** | **p-value** |
| --- | --- | --- | --- | --- | --- | --- | --- | --- | --- | --- |
|  | **No** | | **Yes** | | |  | | |  |  |
|  | **n** | **%** | **n** | **%** | **n** | | | **%** |  |  |
| **Allergies** |  |  |  |  |  | | |  | 3.944(2) | 0.139 |
| Yes | 25 | 67.6 | 53 | 84.1 | 78 | | | 78.0 |  |  |
| No | 10 | 27.0 | 9 | 14.3 | 19 | | | 19.0 |  |  |
| Don't know | 2 | 5.4 | 1 | 1.6 | 3 | | | 3.0 |  |  |
| **Asthma** |  |  |  |  |  | | |  | 2.040(2) | 0.361 |
| Yes | 27 | 75.0 | 56 | 83.6 | 83 | | | 80.6 |  |  |
| No | 9 | 25.0 | 10 | 14.9 | 19 | | | 18.5 |  |  |
| Don't know | 0 | 0.0 | 1 | 1.5 | 1 | | | 1.0 |  |  |
| **Cancer** |  |  |  |  |  | | |  | 0.792(2) | 0.673 |
| Yes | 25 | 69.4 | 43 | 64.2 | 68 | | | 66.0 |  |  |
| No | 8 | 22.2 | 20 | 29.9 | 28 | | | 27.2 |  |  |
| Don't know | 3 | 8.3 | 4 | 6.0 | 7 | | | 6.8 |  |  |
| **Cardiovascular disease** |  |  |  |  |  | | |  | 0.730(2) | 0.694 |
| Yes | 35 | 66.0 | 31 | 63.3 | 66 | | | 64.7 |  |  |
| No | 15 | 28.3 | 13 | 26.5 | 28 | | | 27.5 |  |  |
| Don't know | 3 | 5.7 | 5 | 10.2 | 8 | | | 7.8 |  |  |
| **Mental health conditions** |  |  |  |  |  | | |  | 1.255(2) | 0.534 |
| Yes | 27 | 62.8 | 31 | 52.5 | 58 | | | 56.9 |  |  |
| No | 11 | 25.6 | 21 | 35.6 | 32 | | | 31.4 |  |  |
| Don't know | 5 | 11.6 | 7 | 11.9 | 12 | | | 11.8 |  |  |
| **Obesity** |  |  |  |  |  | | |  | 0.768(2) | 0.681 |
| Yes | 37 | 52.1 | 13 | 43.3 | 50 | | | 49.5 |  |  |
| No | 31 | 43.7 | 15 | 50.0 | 46 | | | 45.5 |  |  |
| Don't know | 3 | 4.2 | 2 | 6.7 | 5 | | | 5.0 |  |  |
| **Type 2 diabetes** |  |  |  |  |  | | |  | 0.105(2) | 0.949 |
| Yes | 41 | 66.1 | 25 | 64.1 | 66 | | | 65.4 |  |  |
| No | 19 | 30.7 | 13 | 33.3 | 32 | | | 31.7 |  |  |
| Don't know | 2 | 3.2 | 1 | 2.6 | 3 | | | 3.0 |  |  |
