# Supplementary material for: Australian parental perceptions of genomic newborn screening for non-communicable diseases
Source: Front Genet. 2023 Jun 26;14:1209762. doi: 10.3389/fgene.2023.1209762 (PMC10330815; doi:10.3389/fgene.2023.1209762)
Supplement: Supplementary file 2 [file Table2.DOCX]

**Supplementary table 2.** Effect of having a child with a medical condition on screening preferences for NCDs.

| **Testing Preference** | **Has your child ever had a medical problem?** | | | | **Total** | | **chi2(df)** | **p-value** |
| --- | --- | --- | --- | --- | --- | --- | --- | --- |
|  | **No** | | **Yes** | |  |  |  |  |
|  | **n** | **%** | **n** | **%** | **n** | **%** |  |  |
| **Allergies** |  |  |  |  |  |  | 1.829(2) | 0.401 |
| Yes | 33 | 84.6 | 44 | 74.6 | 77 | 78.6 |  |  |
| No | 5 | 12.8 | 14 | 23.7 | 19 | 19.4 |  |  |
| Don't know | 1 | 2.6 | 1 | 1.7 | 2 | 2.0 |  |  |
| **Asthma** |  |  |  |  |  |  | 2.717(1) | 0.099 |
| Yes | 35 | 89.7 | 46 | 76.7 | 81 | 81.8 |  |  |
| No | 4 | 10.3 | 14 | 23.3 | 18 | 18.2 |  |  |
| **Cancer** |  |  |  |  |  |  | 6.388(2) | **0.041** |
| Yes | 32 | 82.1 | 34 | 57.6 | 66 | 67.4 |  |  |
| No | 6 | 15.4 | 22 | 37.3 | 28 | 28.6 |  |  |
| Don't know | 1 | 2.6 | 3 | 5.1 | 4 | 4.1 |  |  |
| **Cardiovascular disease** |  |  |  |  |  |  | 3.639(2) | 0.162 |
| Yes | 30 | 76.9 | 35 | 58.3 | 65 | 65.7 |  |  |
| No | 7 | 18.0 | 20 | 33.3 | 27 | 27.3 |  |  |
| Don't know | 2 | 5.1 | 5 | 8.3 | 7 | 7.1 |  |  |
| **Mental health conditions** |  |  |  |  |  |  | 3.735(2) | 0.155 |
| Yes | 27 | 69.2 | 30 | 50.9 | 57 | 58.2 |  |  |
| No | 10 | 25.6 | 21 | 35.6 | 31 | 31.6 |  |  |
| Don't know | 2 | 5.1 | 8 | 13.6 | 10 | 10.2 |  |  |
| **Obesity** |  |  |  |  |  |  | 0.963(2) | 0.618 |
| Yes | 22 | 56.4 | 28 | 46.7 | 50 | 50.5 |  |  |
| No | 15 | 38.5 | 29 | 48.3 | 44 | 44.4 |  |  |
| Don't know | 2 | 5.1 | 3 | 5.0 | 5 | 5.1 |  |  |
| **Type 2 diabetes** |  |  |  |  |  |  | 4.725(2) | 0.094 |
| Yes | 28 | 71.8 | 38 | 63.3 | 66 | 66.7 |  |  |
| No | 9 | 23.1 | 22 | 36.7 | 31 | 31.3 |  |  |
| Don't know | 2 | 5.1 | 0 | 0.0 | 2 | 2.0 |  |  |
