# Supplementary material for: Australian parental perceptions of genomic newborn screening for non-communicable diseases
Source: Front Genet. 2023 Jun 26;14:1209762. doi: 10.3389/fgene.2023.1209762 (PMC10330815; doi:10.3389/fgene.2023.1209762)
Supplement: Supplementary file 3 [file Table5.DOCX]

**Supplementary table 5.** Effect of SEIFA score on screening preferences for NCDs

| **Variable** | **SEIFA Quartile** | | | | | | | | **Total** | | **chi2(df)** | **p-value** |
| --- | --- | --- | --- | --- | --- | --- | --- | --- | --- | --- | --- | --- |
|  | **<25% quartile** | | **25% - 50% quartile** | | **51% - 75% quartile** | | **>75% quartile** | |  |  |  |  |
|  | **n** | **%** | **n** | **%** | **n** | **%** | **n** | **%** | **n** | **%** |  |  |
| **Allergies** |  |  |  |  |  |  |  |  |  |  | 4.990(6) | 0.545 |
| Yes | 4 | 80.0 | 12 | 92.3 | 17 | 68.0 | 48 | 80.0 | 81 | 78.6 |  |  |
| No | 1 | 20.0 | 1 | 7.7 | 6 | 24.0 | 11 | 18.3 | 19 | 18.5 |  |  |
| Don't know | 0 | 0.0 | 0 | 0.0 | 2 | 8.0 | 1 | 1.7 | 3 | 2.9 |  |  |
| **Asthma** |  |  |  |  |  |  |  |  |  |  | 7.952(6) | 0.242 |
| Yes | 4 | 80.0 | 13 | 100.0 | 17 | 68.0 | 50 | 82.0 | 84 | 80.8 |  |  |
| No | 1 | 20.0 | 0 | 0.0 | 7 | 28.0 | 11 | 18.0 | 19 | 18.3 |  |  |
| Don't know | 0 | 0.0 | 0 | 0.0 | 1 | 4.0 | 0 | 0.0 | 1 | 1.0 |  |  |
| **Cancer** |  |  |  |  |  |  |  |  |  |  | 7.572(6) | 0.271 |
| Yes | 3 | 60.0 | 10 | 83.3 | 14 | 56.0 | 41 | 67.2 | 68 | 66.0 |  |  |
| No | 2 | 40.0 | 1 | 8.3 | 7 | 28.0 | 18 | 29.5 | 28 | 27.2 |  |  |
| Don't know | 0 | 0.0 | 1 | 8.3 | 4 | 16.0 | 2 | 3.3 | 7 | 6.8 |  |  |
| **Cardiovascular disease** |  |  |  |  |  |  |  |  |  |  | 3.136(6) | 0.792 |
| Yes | 3 | 60.0 | 10 | 76.9 | 14 | 56.0 | 42 | 68.9 | 69 | 66.4 |  |  |
| No | 2 | 40.0 | 2 | 15.4 | 8 | 32.0 | 15 | 24.6 | 27 | 26.0 |  |  |
| Don't know | 0 | 0.0 | 1 | 7.7 | 3 | 12.0 | 4 | 6.6 | 8 | 7.7 |  |  |
| **Mental health conditions** |  |  |  |  |  |  |  |  |  |  | 5.696(6) | 0.458 |
| Yes | 3 | 60.0 | 6 | 46.2 | 13 | 52.0 | 37 | 61.7 | 59 | 57.3 |  |  |
| No | 2 | 40.0 | 3 | 23.1 | 9 | 36.0 | 17 | 28.3 | 31 | 30.1 |  |  |
| Don't know | 0 | 0.0 | 4 | 30.8 | 3 | 12.0 | 6 | 10.0 | 13 | 12.6 |  |  |
| **Obesity** |  |  |  |  |  |  |  |  |  |  | 6.829(6) | 0.337 |
| Yes | 3 | 60.0 | 5 | 38.5 | 12 | 48.0 | 32 | 52.5 | 52 | 50.0 |  |  |
| No | 2 | 40.0 | 5 | 38.5 | 12 | 48.0 | 26 | 42.6 | 45 | 43.3 |  |  |
| Don't know | 0 | 0.0 | 3 | 23.1 | 1 | 4.0 | 3 | 4.9 | 7 | 6.7 |  |  |
| **Type 2 diabetes** |  |  |  |  |  |  |  |  |  |  | 9.704(6) | 0.138 |
| Yes | 3 | 60.0 | 8 | 61.5 | 16 | 64.0 | 43 | 70.5 | 70 | 67.3 |  |  |
| No | 2 | 40.0 | 3 | 23.1 | 8 | 32.0 | 18 | 29.5 | 31 | 29.8 |  |  |
| Don't know | 0 | 0.0 | 2 | 15.4 | 1 | 4.0 | 0 | 0.0 | 3 | 2.9 |  |  |
