# Supplementary material for: Australian parental perceptions of genomic newborn screening for non-communicable diseases
Source: Front Genet. 2023 Jun 26;14:1209762. doi: 10.3389/fgene.2023.1209762 (PMC10330815; doi:10.3389/fgene.2023.1209762)
Supplement: Supplementary file 4 [file Table3.DOCX]

**Supplementary table 3.** Effect of age on screening preferences for NCDs.

| **Testing Preference** | **Age group** | | | | | | | **Total** | | **chi2(df)** | **p-value** |
| --- | --- | --- | --- | --- | --- | --- | --- | --- | --- | --- | --- |
|  | **18 - 34** | | **35 - 44** | | | **45 and over** | |  |  |  |  |
|  | **n** | **%** | **n** | **%** | **n** | | **%** | **n** | **%** |  |  |
| **Allergies** |  |  |  |  |  | |  |  |  | 11.485(4) | **0.022** |
| Yes | 42 | 89.4 | 29 | 64.4 | 10 | | 83.3 | 81 | 77.9 |  |  |
| No | 4 | 8.5 | 15 | 33.3 | 1 | | 8.3 | 20 | 19.2 |  |  |
| Don't know | 1 | 2.1 | 1 | 2.2 | 1 | | 8.3 | 3 | 2.9 |  |  |
| **Asthma** |  |  |  |  |  | |  |  |  | 15.929(4) | **0.003** |
| Yes | 43 | 91.5 | 31 | 68.9 | 11 | | 84.6 | 85 | 81.0 |  |  |
| No | 4 | 8.5 | 14 | 31.1 | 1 | | 7.7 | 19 | 18.1 |  |  |
| Don't know | 0 | 0.0 | 0 | 0.0 | 1 | | 7.7 | 1 | 1.0 |  |  |
| **Cancer** |  |  |  |  |  | |  |  |  | 10.382(4) | **0.034** |
| Yes | 33 | 70.2 | 27 | 60.0 | 8 | | 66.7 | 68 | 65.4 |  |  |
| No | 8 | 17.0 | 18 | 40.0 | 3 | | 25.0 | 29 | 27.9 |  |  |
| Don't know | 6 | 12.8 | 0 | 0.0 | 1 | | 8.3 | 7 | 6.7 |  |  |
| **Cardiovascular disease** |  |  |  |  |  | |  |  |  | 9.480(4) | 0.05 |
| Yes | 35 | 74.5 | 26 | 57.8 | 8 | | 61.5 | 69 | 65.7 |  |  |
| No | 9 | 19.2 | 17 | 37.8 | 2 | | 15.4 | 28 | 26.7 |  |  |
| Don't know | 3 | 6.4 | 2 | 4.4 | 3 | | 23.1 | 8 | 7.6 |  |  |
| **Mental health conditions** |  |  |  |  |  | |  |  |  | 10.958(4) | **0.027** |
| Yes | 33 | 70.2 | 17 | 38.6 | 9 | | 69.2 | 59 | 56.7 |  |  |
| No | 10 | 21.3 | 20 | 45.5 | 2 | | 15.4 | 32 | 30.8 |  |  |
| Don't know | 4 | 8.5 | 7 | 15.9 | 2 | | 15.4 | 13 | 12.5 |  |  |
| **Obesity** |  |  |  |  |  | |  |  |  | 1.837(4) | 0.766 |
| Yes | 26 | 55.3 | 19 | 42.2 | 7 | | 53.9 | 52 | 49.5 |  |  |
| No | 18 | 38.3 | 23 | 51.1 | 5 | | 38.5 | 46 | 43.8 |  |  |
| Don't know | 3 | 6.4 | 3 | 6.7 | 1 | | 7.7 | 7 | 6.7 |  |  |
| **Type 2 diabetes** |  |  |  |  |  | |  |  |  | 15.942(4) | **0.003** |
| Yes | 37 | 78.7 | 24 | 53.3 | 9 | | 69.2 | 70 | 66.7 |  |  |
| No | 10 | 21.3 | 20 | 44.4 | 2 | | 15.4 | 32 | 30.5 |  |  |
| Don't know | 0 | 0.0 | 1 | 2.2 | 2 | | 15.4 | 3 | 2.9 |  |  |
