# Supplementary material for: Australian parental perceptions of genomic newborn screening for non-communicable diseases
Source: Front Genet. 2023 Jun 26;14:1209762. doi: 10.3389/fgene.2023.1209762 (PMC10330815; doi:10.3389/fgene.2023.1209762)
Supplement: Supplementary file 5 [file Table4.DOCX]

**Supplementary table 4.** Effect of education on screening preferences for NCDs

| **Variable** | **Education** | | | | | | **Total** | | **chi2(df)** | **p-value** |
| --- | --- | --- | --- | --- | --- | --- | --- | --- | --- | --- |
|  | **High school/ College certificate/ Diploma** | | **Undergraduate degree** | | **Postgraduate degree** | |  |  |  |  |
|  | **n** | **%** | **n** | **%** | **n** | **%** | **n** | **%** |  |  |
| **Allergies** |  |  |  |  |  |  |  |  | 12.473(4) | **0.014** |
| Yes | 13 | 92.9 | 29 | 93.6 | 35 | 66.0 | 77 | 78.6 |  |  |
| No | 1 | 7.1 | 1 | 3.2 | 17 | 32.1 | 19 | 19.4 |  |  |
| Don't know | 0 | 0.0 | 1 | 3.2 | 1 | 1.9 | 2 | 2.0 |  |  |
| **Asthma** |  |  |  |  |  |  |  |  | 10.736(2) | **0.005** |
| Yes | 14 | 100.0 | 29 | 93.6 | 38 | 70.4 | 81 | 81.8 |  |  |
| No | 0 | 0.0 | 2 | 6.5 | 16 | 29.6 | 18 | 18.2 |  |  |
| **Cancer** |  |  |  |  |  |  |  |  | 14.076(4) | **0.007** |
| Yes | 11 | 84.6 | 22 | 71.0 | 33 | 61.1 | 66 | 67.4 |  |  |
| No | 2 | 15.4 | 5 | 16.1 | 21 | 38.9 | 28 | 28.6 |  |  |
| Don't know | 0 | 0.0 | 4 | 12.9 | 0 | 0.0 | 4 | 4.1 |  |  |
| **Cardiovascular disease** |  |  |  |  |  |  |  |  | 5.387(4) | 0.25 |
| Yes | 10 | 71.4 | 22 | 71.0 | 33 | 61.1 | 65 | 65.7 |  |  |
| No | 2 | 14.3 | 6 | 19.4 | 19 | 35.2 | 27 | 27.3 |  |  |
| Don't know | 2 | 14.3 | 3 | 9.7 | 2 | 3.7 | 7 | 7.1 |  |  |
| **Mental health conditions** |  |  |  |  |  |  |  |  | 8.101(4) | 0.088 |
| Yes | 11 | 78.6 | 21 | 67.7 | 25 | 47.2 | 57 | 58.2 |  |  |
| No | 2 | 14.3 | 6 | 19.4 | 23 | 43.4 | 31 | 31.6 |  |  |
| Don't know | 1 | 7.1 | 4 | 12.9 | 5 | 9.4 | 10 | 10.2 |  |  |
| **Obesity** |  |  |  |  |  |  |  |  | 10.938(4) | **0.027** |
| Yes | 11 | 78.6 | 18 | 58.1 | 21 | 38.9 | 50 | 50.5 |  |  |
| No | 3 | 21.4 | 10 | 32.3 | 31 | 57.4 | 44 | 44.4 |  |  |
| Don't know | 0 | 0.0 | 3 | 9.7 | 2 | 3.7 | 5 | 5.1 |  |  |
| **Type 2 diabetes** |  |  |  |  |  |  |  |  | 9.568(4) | **0.048** |
| Yes | 11 | 78.6 | 24 | 77.4 | 31 | 57.4 | 66 | 66.7 |  |  |
| No | 2 | 14.3 | 6 | 19.4 | 23 | 42.6 | 31 | 31.3 |  |  |
| Don't know | 1 | 7.1 | 1 | 3.2 | 0 | 0.0 | 2 | 2.0 |  |  |
