# Supplementary material for: Australian parental perceptions of genomic newborn screening for non-communicable diseases
Source: Front Genet. 2023 Jun 26;14:1209762. doi: 10.3389/fgene.2023.1209762 (PMC10330815; doi:10.3389/fgene.2023.1209762)
Supplement: Supplementary file 6 [file DataSheet1.DOCX]

**SUPPLEMENTARY DATA – QUESTIONNAIRE**
