# Supplementary material for: Australian parental perceptions of genomic newborn screening for non-communicable diseases
Source: Front Genet. 2023 Jun 26;14:1209762. doi: 10.3389/fgene.2023.1209762 (PMC10330815; doi:10.3389/fgene.2023.1209762)
Supplement: Supplementary file 7 [file Image1.PDF]

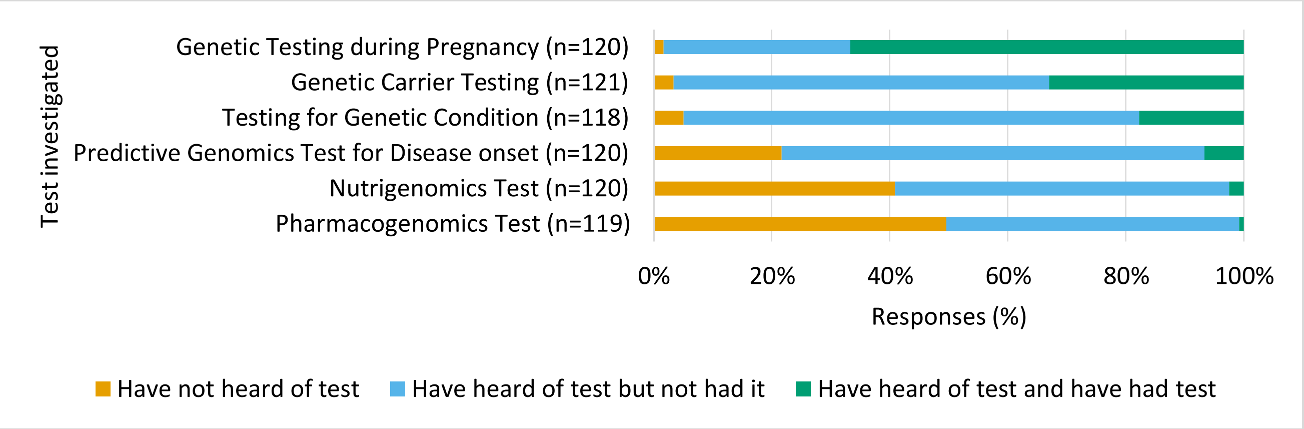

Supplementary Figure 1. Participants' and/or their children's experiences with various genetic tests. The most recognised test was genetic testing during pregnancy, for which 98.3% of participants had heard of the test and 66.7% had the test. Pharmacogenomic testing was the least recognised test, with only 50.4% of participants having heard about the test and only 0.8% of participants having undertaken the test.
